# Assessment of Hand Grip and Pinch Strengths in Children with Autism Spectrum Disorders: A Cross-Sectional Study

**DOI:** 10.3390/children12030320

**Published:** 2025-03-01

**Authors:** Olfat Ibrahim Ali, Hassan Izzeddin Sarsak, Malak Mohammed Tarshi, Manar Marji, Shmookh Turki Aljohani, Maryam Nayef Badawood, Mawda Mohammed Bamusallam, Khawla Alharbi, Zizi M. Ibrahim, Bodor Bin Sheeha, Wafaa Mahmoud Amin

**Affiliations:** 1 Physical Therapy Program, Batterjee Medical College, Jeddah 21442, Saudi Arabia; 2 Basic Science for Physical Therapy Department, Faculty of Physical Therapy, Cairo University, Giza 12613, Egypt; wafaa_770@yahoo.com; 3 Occupational Therapy Program, Batterjee Medical College, Jeddah 21442, Saudi Arabia; hassan.sarsak@bmc.edu.sa (H.I.S.); 150263.malak@bmc.edu.sa (M.M.T.); 5206.manar@bmc.edu.sa (M.M.); 150058.shmookh@bmc.edu.sa (S.T.A.); 21110734@bmc.edu.sa (M.N.B.); 21110002@bmc.edu.sa (M.M.B.); 140162.khawla@bmc.edu.sa (K.A.); 4 Department of Rehabilitation Sciences, College of Health and Rehabilitation Sciences, Princess Nourah bint Abdulrahman University, P.O. Box 84428, Riyadh 11671, Saudi Arabia; zmibrahim@pnu.edu.sa (Z.M.I.); bhbinsheeha@pnu.edu.sa (B.B.S.); 5 Department of Physical Therapy, College of Nursing and Health Sciences, Jazan University, Jazan 45142, Saudi Arabia

**Keywords:** autism, hand strength, pinch strength, handheld dynamometer, pinch gauge

## Abstract

**Background/objective:** Children with autism spectrum disorders (ASD) are recognized to experience challenges with muscle function. However, there is a lack of knowledge about muscle and hand grip strength in autism. Therefore, the study aims to assess the handgrip and pinch strength of ASD and typical children. **Material and method:** This study enrolled 45 participants of both sexes, 27 males and 18 females, aged 6–12 years old; 24 (13 females and 11 males) were typically developing children and 21 (5 females and 16 males) had ASD. The outcome measures were hand grip and pinch strength, with the handheld dynamometer assessing handgrip strength and the hydraulic pinch gauge evaluating pinch grip. The hydraulic pinch gauge measured the pinch strengths of the key, tripod, and pulp grips. Both groups were assessed on both their dominant and non-dominant sides. **Result:** A comparison of the ASD group with the control group revealed that children with ASD exhibited lower values of the hand grip, key pinch, tripod pinch, and pulp pinch strengths for both hands (*p* < 0.05). **Conclusions:** The hand and pinch strengths of ASD children are lower than those of typically developing children. Therefore, the evaluation process for children with ASD should include hand and pinch strengths as a standard component. Furthermore, the rehabilitation program for ASD should prioritize enhancing hand strength.

## 1. Introduction

As defined by the Diagnostic and Statistical Manual of Mental Disorders, autism spectrum disorder (ASD) is characterized by three primary attributes: qualitative deficits in social interaction, communication impairments, and restricted, repetitive, and stereotyped patterns of behavior, interests, and activities [1]. In addition to the three core features of ASD, studies have demonstrated that it is associated with a range of other health dysfunctions [2,3], including physical issues affecting gross and fine movement [4], balance [5], motor and functional skills [6], gait patterns [7], posture, and muscle tone [8], causing hypotonia [9]. Researchers have identified a reduction in skeletal muscle strength as a potential indicator of physical difficulties associated with ASD. A limited number of studies have suggested that the skeletal muscles of children with ASD may be weaker than those of typically developing children of a similar age, gender, and ethnicity [10].

The prevalence of ASD is increasing globally over time. Overall, there are 98 cases of ASD per 10,000 children, with a higher prevalence in boys than in girls and a higher prevalence in developing countries compared to developed ones [11]. However, most scientific studies on ASD have been conducted in developed countries, where comprehensive support services are more easily accessible [12]. Future research should be conducted in developing countries to design appropriate health and social care services. Autism prevalence in Saudi Arabia is slightly higher than in developed countries. In 2002, it is estimated that there are around 42,500 diagnosed cases of ASD, with many unidentified [13].

It is estimated that over 90% of individuals with ASD typically present with additional medical concerns [14]. These include a prevalence of movement disabilities (79%), gastrointestinal diseases (70%), sleep disturbances (50–80%), and intellectual difficulties (45%) [15]. Previous studies have shown that 85% of children with ASD struggle with fine and gross motor skills, especially between the ages of 5 and 15 [16,17]. In a 2021 study by Nordine et al., the development of children with ASD aged 1 to 5 was assessed using the Schedule of Growing Skills II (SGS II). This study revealed a more pronounced delay in fine motor skills and significant delays in gross motor skills [18]. Another study examined the connection between executive functions and motor skills in children with ASD aged 6 to 12. The findings indicated that these children demonstrated significantly lower motor performance than their typically developing peers. The same study also revealed a strong association between motor skills and executive functions [19].

Individuals with ASD encounter difficulties in various domains of their lives. Among preschool and school-aged children with ASD, one prevalent challenge is the disruption of activities and behaviors, including the use of peculiar or repetitive finger and hand gestures, a proclivity for consistency, and a tendency to struggle with adaptation to change and repetitive play with toys (e.g., lining them up, flicking on and off lights) [20]. Hand and pinch grip strength are essential for daily activities, including eating, brushing, and combing [1]. Hand grip strength is essential to child development, enhancing motor skills and self-reliance in various activities. Furthermore, hand grip strength plays a role in school-related activities, such as playing with peers, enhancing handwriting legibility, developing pencil control, and fostering independence in performing functional, delicate motor tasks [21]. Low muscle tone and muscle weakness are associated with motor impairments in children with ASD. Bhat et al. (2011) indicated that children with ASD exhibit constraints in daily tasks, such as reaching and gait, which may be attributable to weakness and atypical tone of the skeletal muscles. Moreover, the researchers suggested deficiencies in supination and pronation, as demonstrated by actions such as twisting a bottle cap or turning a doorknob, may serve as an initial indicator of motor dysfunction in individuals with ASD [22]. The extant literature must consistently assess hand grip and pinch grip strengths. Kern et al. (2013) evaluated the hand muscle strength of 37 children with ASD using a handheld dynamometer. This study’s findings demonstrated a correlation between the severity of impairment and a decline in the child’s hand grip strength [23]. However, Alaniz et al. (2015) observed no significant difference in grip and pinch strength between children with ASD and those without the disorder in a subsequent study [21]. Age, gender, and ethnicity closely correlate with anthropometric measurements, particularly handgrip and pinch grip strengths in children and young adults. In light of these findings, numerous countries have established databases for the norms of anthropometric measurements among the different strata of their population. In developed nations, handgrip and pinch grip strength were more significant in males [24]. The norms of strength for handgrip and pinch grip in healthy children and adolescents (6–18 years old) in Saudi Arabia demonstrated a direct correlation with age. Males exhibited greater grip and pinch grip strength than females [25]. However, limited studies were conducted to evaluate the strength of handgrip and pinch grip in children with disabilities, particularly in children with ADS. Furthermore, as the prevalence of ASD is on the rise [26], healthcare professionals must be more cognizant of the sensory, motor, and perceptual discrepancies commonly observed in children with the condition. Early identification of hand problems enables the prompt implementation of intervention strategies and facilitates the integration of these children into society. The objective of this study was to assess handgrip and pinch strengths in children with ASD in the KSA and to compare hand and pinch strengths between children with ASD and typically developing children.

## 2. Materials and Methods

### 2.1. Study Design and Setting

This cross-sectional descriptive study included 45 schooled children, comprising 21 children with ASD and 24 healthy developing children. We recruited the ASD children from the Saudi Autistic Society in Jeddah, Saudi Arabia, between January and May 2024. 

### 2.2. Participants

ASD individuals were diagnosed by pediatric neurologists and clinical psychologists depending on the DSM-5 (Diagnostic and Statistical Manual of Mental Disorders, 5th edition). ASD children who met the following criteria were eligible to participate in the study: Children of both sexes, their ages ranged from 6 to 12 years old, receiving occupational therapy, physical therapy, and speech therapy. In the current study level one and two were included according to the DSM-5 ASD levels of severity: level 1 requiring support, and level 2 requiring substantial support [27]. Concerning the inclusion criteria of the typically developing children who acted as the control group, each sex was enrolled with ages 6–12 years old. We excluded ASD or neurotypical children exhibiting any of the following criteria: (1) cardiopulmonary and metabolic diseases, which can cause muscle weakness and fatigue, potentially affecting grip and pinch strength measurements [27,28]; (2) prior surgeries or fractures in the upper extremities, which could impair hand function and distort strength assessments [28]; (3) blindness, as vision is important for motor coordination, and its absence could hinder reliable test performance [27]; (4) mental or neurological disorders, which might interfere with the ability to follow instructions and perform tasks accurately [29]; (5) experiencing pain during assessment, as pain can reduce effort and lead to inaccurate strength measurements [30]; and (6) refusal to participate, since unwillingness could result in unreliable data due to lack of effort. Moreover, children in the control group were excluded if they engaged in any sports activities. These criteria were established to ensure that the findings accurately reflect the grip and pinch strength characteristics of the participants. 

All children agreed to participate orally, and the caregivers signed a consent form outlining the study’s aims and procedures before enrollment. The institutional review board of the Batterjee Medical College approved the study under reference number RES-2024-0011.

### 2.3. Sample Size Calculation

The requisite sample size was determined based on hand grip strength, the primary outcome of this investigation. The calculation was conducted utilizing G*Power 3.1 software (Universities, Düsseldorf, Germany). The significance level was set at 5%, the statistical power was 80%, and the effect size was 0.8 according to Cohen’s d. This resulted in a total sample size of 19 participants for each group.

### 2.4. Measured Outcomes

#### 2.4.1. Handgrip Strength

The primary outcome measure was handgrip strength, which was assessed using a handheld dynamometer. This device measures the strength of maximum isometric contraction of the muscles of the hand and forearm. It is a valid, reliable, accurate, and objective tool for identifying variations in hand muscle strength. The device utilizes kilograms (kg) and pounds (lbs) to determine the measurement [28,29].

#### 2.4.2. Pinch-Grip Strength

The second outcome measure was pinch-grip strength, which includes key, tripod, and pulp pinches. The hydraulic pinch gauge, which consists of a two-fingered power measurement tool and a high-speed gauge, assessed this. A hydraulic pinch gauge is valid and reliable for measuring pinch grip strengths. It measures the compressive force exerted by two fingers and transmits the measured values to a high-velocity gauge to quantify the force. The calculation unit is either kilograms (kg) or pounds (lbs) [30].

### 2.5. Assessment Procedures

#### 2.5.1. Assessment of Hand Grip Strength

**Figure 1:**
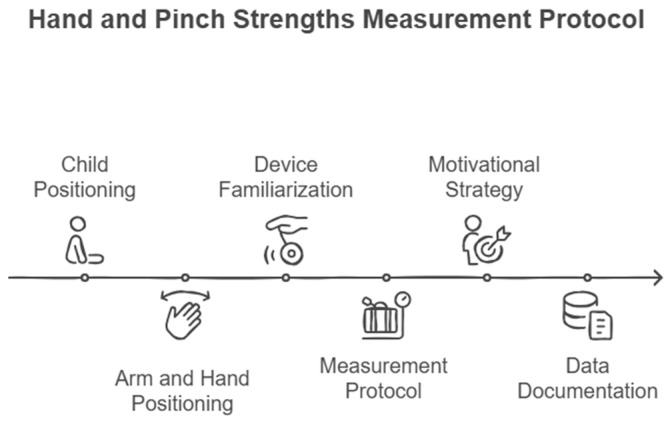
Schematic diagram of the hand and pinch grip strengths assessment procedure.

**Figure 2:**
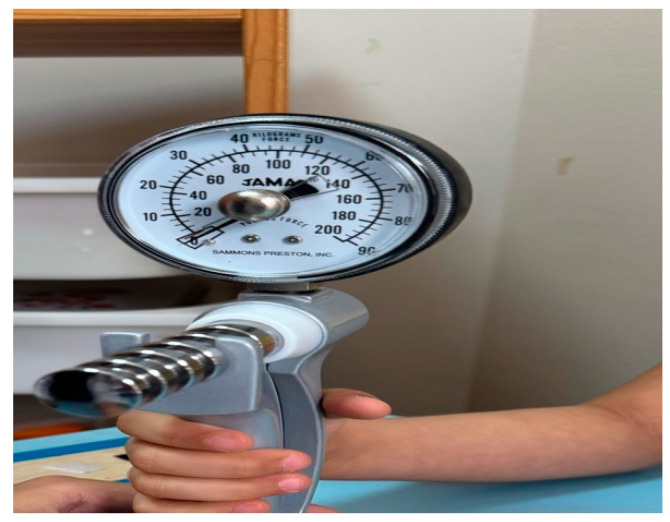
Measuring hand grip strength of ASD children.

The child was positioned on a chair with back support in front of a table on which the handheld dynamometer was placed. The trunk and thigh were at a right angle, with the feet supported on the ground. The child gripped the device handle while the arm was maintained in the supporting cast of the handheld dynamometer. The standardized clinical assessment arm position recommended by the American Society of Hand Therapists was adduction and neutral rotation position of the shoulder, 90° flexion of the elbow, semi-pronation position of the forearm, and neutral wrist position. We placed pleated cloth beneath the forearm of younger children to prevent extensive radial deviation. Anti-slip material was placed underneath the handheld dynamometer’s platform to support it and prevent sliding as presented in Figure 1. The examiner utilized a stabilizing technique to secure the platform. To familiarize themselves with the device, the child grasped the handle, established a secure grip, applied a slight pressure, and observed a corresponding rise in grip strength on the digital monitor, as shown in Figure 2. After preparing the child, we instructed them to squeeze with maximum force for 10 s, starting with the verbal cue “go”. We took alternating measurements between the hands, usually starting with the dominant one, and recorded three trials for each hand, ensuring a minimum of a two-minute interval between each trial. A test protocol documented the results of each test. To ensure consistent effort across all participants, we employed a reward-based motivational strategy. After completing each assessment, participants received small tangible rewards, such as stickers or tokens. This approach was used in conjunction with verbal encouragement to enhance engagement and effort, particularly for children with ASD. Reward-based motivation has been shown to be effective in promoting participation and task performance in children with ASD [28,31,32,33,34].

#### 2.5.2. Assessment of Pinch Strength

As the American Society of Hand Therapists outlined, the pinch strength of each individual’s left and right hand was assessed [35,36]. The child was seated in a chair with 90 degrees of elbow flexion, forearms in a neutral position, shoulders adducted, and neutrally rotated. The wrist was flexed from 0 to 30 degrees, with an ulnar deviation of approximately 0 to 15 degrees. After explaining the measurement process, the rater verbally instructed the subjects to exert their maximal strength for three to five seconds. After completing three consecutive tests of both hands, we calculated the mean values in Figure 1 [30].

**Figure 3:**
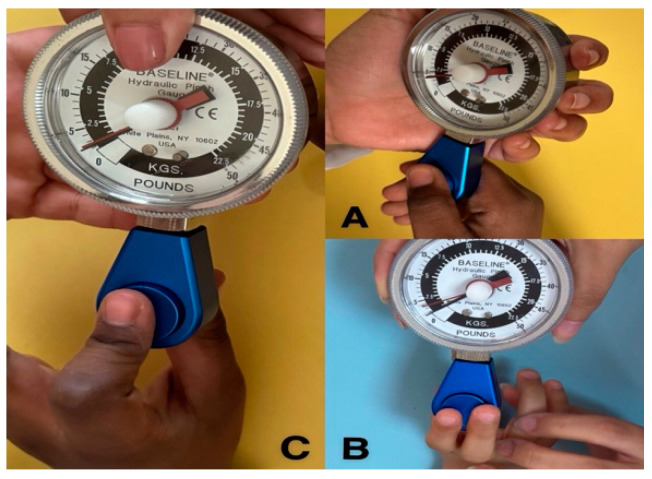
Measuring pinch strengths of ASD children (**A**) key pinch, (**B**) tripod pinch, and (**C**) pulp pinch.

We quantified the key pinch by measuring the pressure between the thumb pad’s palmar surface and the index finger’s lateral side, as shown in Figure 3A. Figure 3B illustrates the tripod pinch between the palmar surfaces of the index and middle fingers and the thumb. Figure 3C illustrates the measurement of the pulp pinch between the thumb’s palmar surface and the index finger. The researchers provided minimal support at the base and the pinch gauges at the distal end to prevent the dynamometer from falling. Hand dominance was determined based on the child’s demonstrated preference for the dominant hand in daily tasks. Before commencing the measurement of hand strengths, all children were required to be familiarized with handheld and pinch dynamometers to ensure a satisfactory understanding of the procedures. Regular calibration of the dynamometers was conducted to guarantee the precision and dependability of the grip and pinch strength measurements [37].

### 2.6. Data Analysis

The demographic variables, including sex, age, weight, height, body mass index (BMI), and the specific type of ASD, were documented. The participant’s weight and height were recorded in kilograms (kg) and centimeters (cm); accordingly, BMI was computed as the ratio of weight to the square of height (kg/m^2^). A *t*-test was employed to compare demographic data, while a chi-square test was utilized to compare the sexes. ANCOVA (Analysis of Covariance) test was utilized to compare hand grip and pinch strengths between ASD and children with typical development controlling for age, sex and body mass index (BMI) as a covariate. Given the nonparametric distribution of the numerical data, we conducted a Mann–Whitney U test for a between-group comparison of the outcome measures. The mean ± standard deviation (SD) was employed to represent the numerical data, whereas the numbers and percentages (*n*, %) were utilized to describe the nominal data. The final statistical analysis was conducted using SPSS version 29 (SPSS Inc., Chicago, IL, USA) for Windows^®^.

## 3. Results

### 3.1. Demographic Data of the Children

Table 1 presents a comparative analysis of the characteristics of both groups. No significant differences were observed between the two groups regarding age, weight, height, BMI, and sex, as indicated by *p*-values exceeding 0.05.

Results of analysis of covariate revealed a statistically significant difference between groups of the tested outcomes (Rt and Lt hand grip strength, key pinch, tripod pinch and pulp pinch strengths) after adjusting the effect of age (*p* < 0.05), sex (*p* < 0.05) and BMI (*p* > 0.05) (Table 2) 

### 3.2. Concerning Handgrip Strength

Table 3 indicates that children with ASD demonstrated handgrip strength values of 1.73 ± 1.62 kg for the right hand and 1.32 ± 1.67 kg for the left hand. In comparison, typically developing children demonstrated handgrip strength values of 6.66 ± 4.54 kg for the right hand and 6.48 ± 4.16 kg for the left. A statistically significant reduction in the grip strength of both hands was found in children with ASD when compared to their peers of the same gender and age (*p* < 0.001). These findings indicate that ASD children showed weaker hand grip force compared to typically developed children.

### 3.3. Concerning Pinch Strengths

The mean pinch strength values for the right hand in children with ASD were 2.88 ± 1.09, 2.78 ± 1.05, and 2.16 ± 0.66 for the key, tripod, and pulp pinches, respectively. In contrast, the pinch strengths of the right hand of children in the neurotypical group were 4.64 ± 1.43, 4.33 ± 1.23, and 3.10 ± 0.94. The left-hand pinch strengths of children with ASD were 3 ± 1.03, 2.82 ± 0.91, and 1.98 ± 0.63. However, the values of the left pinch strengths of the neurotypical children exhibited mean pinch strengths of 4.18 ± 1.26, 4.05 ± 1.10, and 3.07 ± 1.02 for the key, tripod, and pulp pinch, respectively. The preceding findings indicate a statistically significant variation in right and left pinch strengths between neurotypical children and children with ASD (*p* < 0.001). In contrast to typical children, children with ASD exhibited poor pinch strengths, as illustrated in Table 3. 

### 3.4. Comparison Between ASD Levels One and Two

A comparison of the ASD levels revealed no statistically significant difference between levels one and two regarding demographic data (age, weight, height, and BMI) where the *p*-value is less than 0.05. Moreover, the Rt and Lt hand grip, key pinch, tripod pinch, and pulp pinch strengths demonstrated no statistical difference as *p* values were (0.736, 0.486, 0.971, 0.495, 0.479, 0.193, 0.232, 0.731), respectively (Table 4).

## 4. Discussion

The handgrip and pinch strengths are crucial in children’s lives, particularly during the school-age years, which were the focus of this research. The development of other children’s life skills, activities of daily living (ADLs), and instrumental ADLs (IADLs), such utilizing a telephone, which is now an essential tool for engaging and communicating with society, depends on the strength of the handgrip and pinch grip [38].

This study evaluated three types of pinches regarding their functional significance and priority in children’s daily activities. The key or lateral pinch is utilized in many activities, including self-feeding with utensils such as spoons and forks and grasping keys to open doors, even in the context of children’s games. The tripod pinch is employed in a variety of educational and writing activities, as well as in the act of grasping objects. The pulp pinch is the most refined of the three. People use this pinch to pick up small objects and turn pages of books [39].

The results of the current study showed that, in comparison to typically developing children, children with ASD have poor handgrips and pinch grip strength. The pulp pinch was the weakest pinch grip in ASD children, followed by the tripods and the key pinch, in that order. In addition, there were no significant differences between ASD levels one and two in assessing demographic characteristics and all measured outcomes. These findings suggest that this weakness may significantly affect other aspects of children’s ADLs, IADLs, and functional life activities. The current findings of weak strengths among ASD children might be attributed to cognitive limitations such as problems in executive function [40], muscle weakness [22], coordination problems [41], and difficulties in pronation and supination that ASD children may experience [42].

Children with ASD have unique motor behavior, including motor stereotypies, coordination, and control variations [43,44], which are manifested by delays in both motor milestones [45], and gross and fine motor functions in childhood and may extend to adulthood [46,47]. The DSM-5, and International Classification of Diseases, 11th Edition guidelines have studied and identified the co-occurring developmental motor coordination disorder (DCD) and motor problems in children with ASD. Some studies related the deficiency in motor performance in children with ASD to the overlap with DCD in early development [44,48], and others related it to the sensorimotor interaction mechanism [44]. Studies have shown that children with ASD have atypical or unusual sensory reactions in the form of hypo- and hyper-reactive responses that affect their skills of self-care and academic performance [43,44,45,46]. Also, they interfere with engagement in family daily routines and practicing motor activity [49]. They have sensory–motor integration disorders resulting from structural abnormalities of the cortical-cerebellar network. These disorders lead to motor incoordination, poor balance, and improper postural control [50].

Our study’s findings align with those of Kern et al. (2013), who conducted a similar study in Texas on children of the same age. They hypothesized and confirmed that children with ASD exhibit a greater reduction in handgrip strength compared to children with typical development peers [23]. Moreover, Fuentes et al. (2009) discovered that children with ASD exhibited diminished overall performance in handwriting skills relative to their age-matched controls [51].

Previous studies have investigated motor impairment in both fine and gross motor function in ASD [9,16,22], which may elucidate the current findings of this study. Ming et al. (2007) posited that motor impairment is highly prevalent among children with ASD, particularly in the form of hypotonia [9]. Bhat et al. (2011) observed that children with ASD exhibited general motor impairments, including muscle tone disturbance, weakness of skeletal muscles, and loss of coordination during gross and fine motor activities. These impairments led to limitations in ADLs, such as stair climbing, gait, and reaching [22]. Furthermore, Bhat (2020) determined that children with ASD are at an 86.9% increased risk of developing motor impairments, such as developmental coordination disorder [16].

The variability of motor impairments across autism severity levels denotes an essential aspect of comprehending the functional impact of ASD. According to the DSM-5; ASD severity was classified into three levels, indicating the amount of support needed and the ASD functional impairment. However, these levels are not fixed and are subjected to changes over time according to the children’s development, surrounding environment, and any Intervention delivered to those children [27].

The present research found no significant difference in muscle strength between children classified as ASD Level 1 and Level 2. This aligns with evidence suggesting that motor impairments in ASD are more likely attributable to broader sensorimotor integration deficits rather than severity level alone [44].

In contrast to the present finding that revealed no significant difference between ASD levels one and two, Kern et al. (2011) evaluated handgrip strength using a handheld dynamometer based on the severity of ASD. The researchers concluded that children with ASD who exhibited a high degree of severity demonstrated weaker hand strength [52]. This contradiction could be explained by Kern et al. study includes mild, moderate, and severe types according to the Childhood Autism Rating Scale (CARS) classification however our research includes levels one and two only according to the DSM-5.

Moreover, Yamaguchi et al. (2019) reported that children with ASD also exhibited impaired hand functions, including supination and pronation, repetitive hand tapping, finger lifting, sequential tapping of the finger, and pegboard performance [42].

The present findings align with those of Chen et al. (2019), who demonstrated that the motor difficulties associated with ASD may originate from impaired coordination between sensory and motor processes during movement planning and control. This sensory–motor incoordination can manifest as difficulties in activities such as ball catching, which may subsequently impact the child’s occupational performance [41].

The findings of Ludyga et al. (2021) support the present findings. Their study examined the correlation between ASD, muscle strength, and BMI with the capacity to execute functions and process information. To this end, they assessed muscle strength in some selected activities, including grip strength, and employed National Institutes of Health (NIH) Toolbox cognitive tests to evaluate the ability to execute functions and process information. The researchers discovered that ASD is significantly associated with diminished muscle strength and impaired function execution and information processing. Additionally, muscle strength exerted an independent influence on function execution and information processing in children with ASD, with more significant improvement in executive function observed as muscle strength increased. This was the first evidence that enhancing muscle strength through regular exercise could reduce the executive dysfunction associated with ASD [40].

However, Alaniz et al. (2015) found no significant differences in hand grip and pinch strengths between ASD and neurotypical children, suggesting that variations in muscle performance may depend on factors such as sample characteristics, age range, or the severity of ASD [21].

A notable strength of present study was the use of a reward-based motivational strategy to address potential variability in effort between children with ASD and their typically developing peers. Evidence suggests that tangible rewards are particularly effective in engaging children with ASD, as they may not respond consistently to verbal encouragement. While this strategy likely reduced variability in effort, future studies should further explore additional methods to standardize motivation across groups, such as gamification or visual cues.

### Limitations of the Study

The current study has certain limitations: Firstly, the sample was restricted to children aged 6 to 12, and future studies would benefit including participants from a wider range of age groups. Secondly, although we incorporated reward-based motivation to encourage consistent effort, the variability in how children with ASD and their typically developing peers respond to motivational strategies could still have influenced the results. Future research should investigate alternative approaches to standardize effort further, such as incorporating gamified tasks or objective measures of engagement. Thirdly, the study only included three types of pinch grips, indicating the need for additional research to assess additional types. Furthermore, the current study did not account for participants’ socioeconomic background, variations in physical activity levels, or nutritional differences. Future research should consider these factors, along with children’s physical activity levels and overall lifestyle. Additionally, further studies utilizing standardized assessment tools are necessary to evaluate the impact of weak grip strength on children’s functional activities. Finaly, given the relatively small sample size of this study, future research should include a larger sample to enhance the reliability and generalizability of the findings.

## 5. Conclusions

This study showed a significant difference in handgrip strength and pinch grip strength between ASD and children with typical development. The findings suggest that children with ASD exhibit diminished and impaired handgrip and pinch grip strength compared to their typically developing counterparts. Future rehabilitation programs should prioritize enhancing handgrip and pinch grip strength in children with ASD. Additionally, the implementation of compensatory strategies to facilitate engagement in daily life activities and improve their developmental process should be considered. We recommend that the joint participation of these children in rehabilitation activities with other children would benefit their functioning in both the physical and psychosocial dimensions.

## Figures and Tables

**Table 1:** Demographic characteristics of children.

Variables	ASD Group	Control Group	*p* Value
	Mean ± SD	Mean ± SD	
Age (yrs)	8.34 ± 2.26	8.6 ± 2.93	0.734
Weight (kg)	30.2 ± 6.46	28.3 ± 10.03	0.236
Height (cm)	129.6 ± 10.4	124.5 ± 18.09	0.435
BMI (kg/m^2^)	17.3 ± 2.1	17.2 ± 3.3	0.921
Gender			
F (43.75%)	5 (23.8%)	13 (54.2%)	0.075
M (56.25%)	16 (76.2%)	11 (45.8%)	
ASD type			
Level I	12 (57.1%)		
Level II	9 (42.9)		

yrs: years, kg: kilogram, cm: centimeter, kg/m^2^: kilogram/meter square, %: percentage, F: female, M: male, ASD: autism spectrum disorder, SD: standard deviation, *p*-value: significance level.

**Table 2:** Analysis of covariance (ANCOVA).

Source		SS	df	MS	F-Value	*p* Value
**Intercept**	Rt Handgrip	10.346	1	10.346	1.299	0.261
	Lt Handgrip	13.389	1	13.389	1.774	0.190
	Rt Key Pinch	5.109	1	5.109	5.281	0.027
	Lt Key Pinch	4.201	1	4.201	5.903	0.020
	Rt Tripod Pinch	5.872	1	5.872	12.055	0.001
	Lt Tripod Pinch	1.698	1	1.698	3.406	0.072
	Rt Pulp Pinch	1.832	1	1.832	4.264	0.045
	Lt Pulp Pinch	0.946	1	0.946	1.551	0.220
**Gender**	Rt Handgrip	0.628	1	0.628	0.079	0.780
	Lt Handgrip	2.240	1	2.240	0.297	0.589
	Rt Key Pinch	3.982	1	3.982	4.116	0.049
	Lt Key Pinch	2.365	1	2.365	3.324	0.076
	Rt Tripod Pinch	8.137	1	8.137	16.704	0.000
	Lt Tripod Pinch	2.139	1	2.139	4.290	0.045
	Rt Pulp Pinch	1.733	1	1.733	4.033	0.051
	Lt Pulp Pinch	0.084	1	0.084	0.138	0.712
**Age**	Rt Handgrip	251.483	1	251.483	31.568	0.000
	Lt Handgrip	190.776	1	190.776	25.280	0.000
	Rt Key Pinch	39.504	1	39.504	40.833	0.000
	Lt Key Pinch	32.209	1	32.209	45.265	0.000
	Rt Tripod Pinch	34.129	1	34.129	70.061	0.000
	Lt Tripod Pinch	23.530	1	23.530	47.204	0.000
	Rt Pulp Pinch	11.413	1	11.413	26.559	0.000
	Lt Pulp Pinch	8.617	1	8.617	14.128	0.001
**BMI**	Rt Handgrip	0.026	1	0.026	0.003	0.955
	Lt Handgrip	0.146	1	0.146	0.019	0.890
	Rt Key Pinch	1.127	1	1.127	1.165	0.287
	Lt Key Pinch	0.693	1	0.693	0.974	0.330
	Rt Tripod Pinch	0.606	1	0.606	1.244	0.271
	Lt Tripod Pinch	0.061	1	0.061	0.123	0.727
	Rt Pulp Pinch	0.000	1	0.000	0.001	0.982
	Lt Pulp Pinch	0.004	1	0.004	0.006	0.937
**Groups**	Rt Handgrip	213.837	1	213.837	26.843	0.000
	Lt Handgrip	272.639	1	272.639	36.128	0.000
	Rt Key Pinch	23.871	1	23.871	24.674	0.000
	Lt Key Pinch	12.257	1	12.257	17.226	0.000
	Rt Tripod Pinch	15.500	1	15.500	31.819	0.000
	Lt Tripod Pinch	12.254	1	12.254	24.583	0.000
	Rt Pulp Pinch	5.796	1	5.796	13.487	0.001
	Lt Pulp Pinch	9.924	1	9.924	16.272	0.000
**Error**	Rt Handgrip	318.652	40	7.966		
	Lt Handgrip	301.862	40	7.547		
	Rt Key Pinch	38.698	40	0.967		
	Lt Key Pinch	28.462	40	0.712		
	Rt Tripod Pinch	19.485	40	0.487		
	Lt Tripod Pinch	19.939	40	0.498		
	Rt Pulp Pinch	17.188	40	0.430		
	Lt Pulp Pinch	24.396	40	0.610		

**Table 3:** Between groups comparison of the mean hand grip and pinch strengths values.

Variables	ASD Group Mean ± SD	Control Group Mean ± SD	*p* Value
Rt Handgrip (kg)	1.73 ± 1.62	6.66 ± 4.54	<0.001
Lt Handgrip (kg)	1.32 ± 1.67	6.48 ± 4.16	<0.001
Rt Key Pinch (kg)	2.886 ± 1.09	4.64 ± 1.43	<0.001
Lt Key Pinch (kg)	3 ± 1.03	4.18 ± 1.26	<0.001
Rt Tripod Pinch (kg)	2.78 ± 1.05	4.33 ± 1.23	<0.001
Lt Tripod Pinch (kg)	2.82 ± 0.91	4.05 ± 1.10	<0.001
Rt Pulp Pinch (kg)	2.16 ± 0.66	3.10 ± 0.94	<0.001
Lt Pulp Pinch (kg)	1.98 ± 0.63	3.07 ± 1.02	<0.001

Rt: right, Lt: left, kg: kilogram, ASD: autism spectrum disorder, SD: standard deviation, *p*-value: significant level.

**Table 4:** Between-comparison of demographic and hand grip and pinch strength values between ASD levels.

Variables	ASD Level One Mean ± SD	ASD Level Two Mean ± SD	*p* Value
Age (year)	8.67 ± 2.7	8.33 ± 1.73	0.690
Weight (kg)	131.75 ± 12.9	127.33 ± 7.55	0.392
Height (cm)	31.67 ± 7.5	29.0 ± 5.57	0.144
BMI (kg/cm^2^)	17.5 ± 1.88	17.33 ± 2.8	0.883
Rt Handgrip (kg)	1.71 ± 1.7	2.0 ± 1.62	0.736
Lt Handgrip (kg)	1.6 ± 2.10	1.16 ± 1.01	0.486
Rt Key Pinch (kg)	2.8 ± 1.19	2.88 ± 1.14	0.971
Lt Key Pinch (kg)	3.14 ± 1.14	2.83 ± 1.031	0.495
Rt Tripod Pinch (kg)	2.92 ± 1.24	2.61 ± 0.93	0.479
Lt Tripod Pinch (kg)	3.1 ± 1.10	2.56 ± 0.64	0.193
Rt Pulp Pinch (kg)	2.29 ± 0.54	1.97 ± 0.85	0.232
Lt Pulp Pinch (kg)	2.13 ± 0.43	1.86 ± 0.88	0.731

Rt: right, Lt: left, kg: kilogram, ASD: autism spectrum disorder, SD: standard deviation, *p*-value: significant level.

## Data Availability

The materials supporting this manuscript are available from the corresponding author upon reasonable request due to privacy and ethical considerations.

## Abbreviations

The abbreviations listed below are used throughout this manuscript.

ASD	Children with autism spectrum disorders
ADLs	Activities of daily living
DSM-5	Diagnostic and Statistical Manual of Mental Disorders, 5th edition
IADLs	Instrumental ADLs
NIH	National Institutes of Health
